# Assessing the effectiveness and safety of liposomal paclitaxel in combination with cisplatin as first-line chemotherapy for patients with advanced NSCLC with regional lymph-node metastasis: study protocol for a randomized controlled trial (PLC-GC trial)

**DOI:** 10.1186/1745-6215-14-45

**Published:** 2013-02-15

**Authors:** Luo Hu, Gong Liang, Wang Yuliang, Zhu Bingjing, Zhou Xiangdong, Xu Rufu

**Affiliations:** 1 Department of Respiratory Diseases, The First Affiliated Hospital of Third Military Medical University, Chongqing, 400038, People's Republic of China; 2 Center of Evidence-Based Medicine and Clinical Epidemiology, The Second Affiliated Hospital of Third Military Medical University, Chongqing, 400038, People's Republic of China

**Keywords:** Liposomal paclitaxel, Cisplatin, Gemcitabine, Regional lymph node metastasis, Trials

## Abstract

**Background:**

Lung cancer is still the leading cause of cancer-related mortality worldwide. Around 80 to 85% of lung cancers are non-small cell lung cancer (NSCLC). Regional lymphatic metastasis is a frequent occurrence in NSCLC, and the extent of lymphatic dissemination significantly determines the prognosis of patients with NSCLC. Hence, identification of alternative treatments for these patients should be considered a priority. Liposomal paclitaxel is a new formulation composed of paclitaxel and liposomes, with favorable pharmacokinetic properties. In particular, it produces dramatically higher drug concentrations in the lymph nodes than occurs with the current formulations of paclitaxel, thus we believe that patients with NSCLC with regional lymphatic metastasis may benefit from this new drug. Cisplatin-based doublet chemotherapy is recommended as the first-line treatment for patients with advanced NSCLC. We have designed a trial to assess whether first-line chemotherapy using liposomal paclitaxel combined with cisplatin (LP regimen) is superior to gemcitabine combined with cisplatin (GP regimen) in efficacy (both short-term and long-term efficacy) and safety (adverse events; AEs).

**Method/Design:**

This is a prospective, open-label, controlled randomized clinical trial (RCT) to assess the therapeutic effects and safety of liposomal paclitaxel. The study aims to enroll 126 patients, who will be randomly allocated to one of the two treatment groups (LP and GP), with 63 patients in each group. Patients will receive four to six cycles of the assigned chemotherapy, and primary outcome will be assessed every two cycles. Patients will be recommended for surgery if the tumor becomes resectable. All participants will be followed up for at least 12 months. The objective response rate (ORR), changes in regional lymphatic metastasis (including number and size) and TNM (tumor, node, metastasis) staging will be the primary outcome measures. Progression-free survival, objective survival, median survival time, 1-year survival rate, toxicity, and time to disease progression will be the secondary outcome measures.

**Conclusions:**

A systematic search has indicated that this proposed study will be the first RCT to evaluate whether liposomal paclitaxel plus cisplatin will have beneficial effects, compared with gemcitabine plus cisplatin, on enhancing ORR, changing TNM staging, improving long-term survival, and reducing the frequency of AEs for patients with NSCLC with regional lymphatic metastasis.

**Trial registration:**

http://www.chictr.org Identifier: ChiCTR-TRC-12602105

## Background

### Regional lymphatic metastasis was generally involved in patients with NSCLC

Lung cancer has become the leading cause worldwide of cancer-related mortality in both men and women [1]. Around 80 to 85% of lung cancers are non-small cell lung cancer (NSCLC), adequate therapeutic planning is largely dependent upon early diagnosis, and surgery is still the preferred treatment for early-stage tumors [2]. However, it is known that approximately 70% of patients with NSCLC have advanced local invasion and/or distant metastasis at the time of diagnosis, and thus surgery is not possible for these patients. Presence of regional lymphatic metastasis is generally found in NSCLC even in the early stages of tumor growth, partly because of the wide distribution of lymphoid tissue in the lung, and also because the extent of lymphatic dissemination also significantly determines the clinical prognosis for patients with NSCLC [3]. Therefore, therapeutic strategies focusing on the issue of regional lymphatic metastasis are of great importance not only for the control of distant lung-cancer metastases, but also for reducing the TNM (tumor, node, metastasis) stage and thus regaining the opportunity for surgery.

Currently, cisplatin-based doublet chemotherapy consisting of cisplatin combined with third-generation drugs (paclitaxel, gemcitabine, vinorelbine, or docetaxel) is commonly recommended as the standard treatment for patients with advanced NSCLC [4-7]. However, the pharmaceutical properties of the drugs or formulations, including poor drug permeability through cell membranes or tissue barriers, short circulating half-life, and rapid metabolism, heavily influence the effectiveness of tumor therapy [8]. Consequently, use of chemotherapy drugs targeting regional lymph nodes remains to be further explored and evaluated.

### The specific pharmaceutical properties of liposomal paclitaxel

Paclitaxel is a complex diterpenoid natural product, which has become a first-line treatment for NSCLC. However, because of its poor water solubility, paclitaxel has been used in an encapsulated form with the organic co-solvents ethanol and polyethoxylated castor oil (a formulation marketed as Taxol®; Bristol-Myers Squibb, New York, NY, USA) for clinical trials, but has been shown to cause toxic effects, including life-threatening anaphylaxis [9]. Liposomal paclitaxel (Lipusu; Luye Pharma Group Ltd., Nanjing, China) is a new formulation of paclitaxel and phosphatidylcholine liposomes. Pharmacokinetic studies in animal models [10-12] have shown that, compared with the current paclitaxel formulation, liposomal paclitaxel has a significantly prolonged elimination half-life and mean retention time, and an apparent larger volume of distribution. Even more unexpected and exciting is that the concentration of liposomal paclitaxel in tissues is dramatically higher than that of paclitaxel, especially in the reticuloendothelial system, including the lymph nodes, liver, and spleen. Several clinical studies [12-17] recently indicated that the efficacy of liposomal paclitaxel equaled or slightly exceeded that of the current paclitaxel (Taxol®) formulation, while having a superior safety profile.

### Hypothesis and study basis

We searched MEDLINE (from 1968 to 2012), EMBASE (from 1977 to 2012), and the Cochrane Library (from 1953 to 2012), using selected key words including ‘liposomal paclitaxel (or paclitaxel liposomes)’, ‘lung cancer (or NSCLC)’, ‘lymphatic metastasis’ and ‘controlled trial (or RCT)’. We did not find any clinical randomized controlled trial (RCT) to date that had examined the role of liposomal paclitaxel in patients with NSCLC with regional lymphatic metastasis. The RCTs performed thus far have focused on patients with advanced NSCLC as a whole. In view of the pharmacokinetic characteristics of liposomal paclitaxel in lymph nodes, we speculate that this new formulation may represent a novel chemotherapy drug, and could play a unique role in the treatment of NSCLC with regional lymphatic metastasis. In this paper, we outline our proposed protocol for a prospective open-label RCT aimed at testing this hypothesis.

### Objective

This clinical trial aims to determine whether first-line therapy using liposomal paclitaxel combined with cisplatin (LP regimen) is more effective and safer than gemcitabine combined with cisplatin (GP regimen) for patients with NSCLC with regional lymphatic metastasis.

## Method/Design

### Study design

This clinical trial is a prospective open-label RCT. The design of the study integrates rigorous, contemporary clinical research methods in accordance with principles set out in the Declaration of Helsinki and the *Guidelines for Good Clinical Practice*. Reporting will be guided by the most recent CONSORT statement [18]. The trial will be comprised of three phases as follows.

Phase 1 (recruitment and assignment of patients). Consenting eligible participants will be recruited in accordance with the inclusion/exclusion criteria and assigned randomly to one of the two treatment groups.

Phase 2 (chemotherapy). Patients will receive four to six cycles of chemotherapy with either LP or GP. Adverse events (AEs) will be rigorously monitored and recorded on a case report form (CRF).

Phase 3 (follow-up). Patients will be followed up for at least 12 months.

### Ethics issue

The study has been approved by the ethics committee of The First Affiliated Hospital of Third Military Medical University (No: KY201006), and is registered at ChiCTR (Chinese Clinical Trial Registry, trial Identifier ChiCTR-TRC-12602105). All study participants will provide written informed consent before participation.

### Subjects

**Figure 1:**
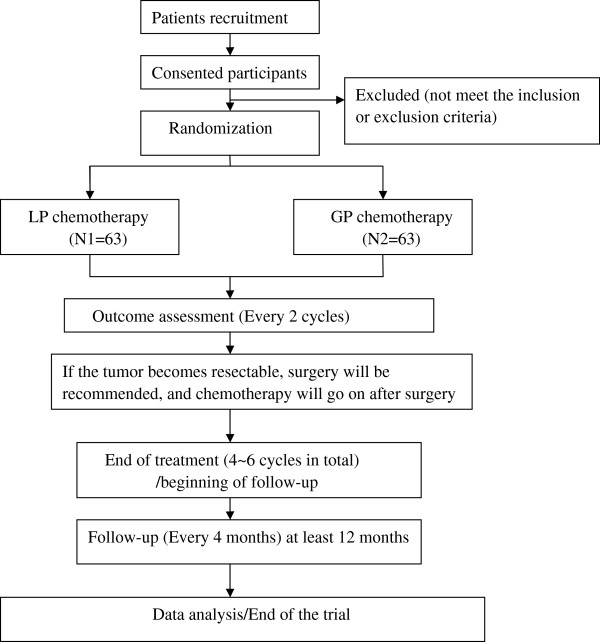
Study flow chart.

The study aims to enroll 126 patients with NSCLC who meet both the inclusion and exclusion criteria below. Using a random-number table (Center of Evidence-Based Medicine and Clinical Epidemiology, The Second Affiliated Hospital, Chongqing), patients will be randomly assigned in a 1:1 ratio to either the LP or the GP group (Figure 1).

### Study criteria

#### Inclusion criteria

Hospitalized patients, both male and female, aged 18 to 75 years.

Eastern Cooperative Oncology Group (ECOG) performance status of 0 to 2.

Pathological diagnosis of non-small cell lung cancer, TNM stage III to IV.

Patients have never received any other chemotherapy.

In accordance with Response Evaluation Criteria in Solid Tumors (RECIST) (RECIST)/WHO evaluation criteria [19], at least one lesion, measurable or evaluable, should be detected by chest CT, and regional lymph-node metastasis should also be measurable or evaluable (including neck and supraclavicular lymph-node metastasis).

Expected survival ≥ 3 months.

Hematology, liver and kidney function are normal, the blood and biochemical indicators should meet the following criteria:

Bone marrow: absolute neutrophil count (ANC) ≥ 1.5 × 10^9^/l (1500/mm^3^)

White blood cell count (WBCC) ≥ 4000 / mm^3^

Platelet count ≥ 100,000 / mm^3^

Hemoglobin ≥ 9 g/dl

Liver: serum bilirubin ≤ 2 times the upper limit of normal (ULN), aspartate aminotransferase (AST) and alanine aminotransferase (ALT) ≤ 2.5 times ULN (or ≤ 5 times normal limit for ALT and AST if liver metastasis present)

Kidney: serum creatinine ≤ 1.5 times ULN.

Good understanding and compliance by patients with the pilot program, and provision of informed consent.

#### Exclusion criteria

Allergy to drugs.

Pregnancy or lactation.

History of cardiovascular disease (CVD), including congestive heart failure (higher than New York Heart Association level II).

Presence of unstable angina (resting angina symptoms) or new onset of angina (in the previous 3 months) or myocardial infarction (in the previous 6 months).

Presence of severe organic disease, including active infection or CVD.

Poor hepatic function or severe cirrhosis.

Abnormal blood coagulation indicators.

Poor condition or cachexia.

Receipt of radiotherapy or other local physical therapy within the previous 6 months.

Receipt of other anti-cancer treatment (such as chemotherapy, radiotherapy, immunosuppressive agents, chemoembolization, or targeted agents).

Recent (1 day to 4 weeks) participation in another clinical drug trial.

Other exclusion criteria: presence of other factors that might lead to failure to participate in or complete the clinical study (such as mental illness or drug abuse), or might affect the analysis of results.

### Study duration

Ongoing recruitment will continue for a maximum of 12 months (between June 2012 and June 2013) or until 126 individuals have been randomly assigned. The full study is expected to be finished before December 2014, when all the patients will have been followed up for at least 12 months.

### Examinations

#### Baseline examinations before treatment

Medical history and demographic information, including sex, age, address, and telephone details.

Physical examination: height, weight, body surface area, and vital signs, including heart rate, blood pressure, respiration rate, and temperature.

Physical status.

Laboratory examinations: routine blood and urine tests, liver and renal function.

Electrocardiography (ECG) and chest computed tomography (CT) scans.

The above examinations should be performed at least 1 week before chemotherapy begins. Tumors should be evaluated on the basis of the tumor-associated symptoms, physical examination of the superficial lesions, and results of imaging studies.

#### Examinations during treatment

Physical status, physical examination, routine blood and urine tests, liver and renal function tests, and ECG should be performed 2 to 3 days before each chemotherapy cycle.

Routine blood tests will be performed on days 4, 8, and 14 after each cycle of chemotherapy. The times will be increased if myelosuppression happens.

During the treatment, tumor will be evaluated 1 week after every two cycles of chemotherapy, as described above.

#### Post-treatment examination

After treatment, participants will be required to return to our department for follow-up every 4 months, at which time repeat chest CT will be performed. Patients will be informed that they should contact us immediately if AEs occur, and in such cases, any relevant examinations needed for treatment will be undertaken.

#### Medical interventions

Based on the manufacturer’s instructions for use of liposomal paclitaxel, pretreatment with intravenous dexamethasone (5 to 10 mg), intravenous cimetidine (300 mg) and intramuscular diphenhydramine (50 mg) should be performed 30 minutes before chemotherapy. The chemotherapy protocol is shown in Table 1.

**Table 1:** Chemotherapy procedure of both groups

**Group**	**Day**	**Intervention**	**Dosage**	**Route of administration**
Treatment (LP)	1	Liposomal paclitaxel	135 to 175 mg/m^2^ in saline (500 ml)^1^	IV drip (over 3 hours)
		Cisplatin	75 mg/m^2^ in saline (500 ml)^2^	IV drip
Control (GP)	1 and 8	Gemcitabine	1000 mg/m^2^ in saline (100 ml)	IV drip (over 30 min)
	1	Cisplatin	75 mg/m^2^in saline (500 ml)^2^	IV drip

Abbreviations: GP, gemcitabine plus cisplatin; IV, intravenous; LP, liposomal paclitaxel.

^1^In the LP group, the liposomal paclitaxel must be used before cisplatin to avoid myelosuppression.

^2^At least 1500 ml of saline should be used for hydration before cisplatin infusion occurs.

The chemotherapy will be given as four to six cycles in total, with every 21 days making up one cycle. One week after every two cycles, the efficacy should be evaluated in accordance with the RECIST criteria [19]. The chemotherapy should go on as long as the efficacy evaluation indicates complete response (CR), partial response (PR), or stable disease. Surgery will be suggested if the TNM stage of the tumor reduces sufficiently for the lesion to become resectable; in such cases, the relevant chemotherapy regimen will continue after surgery.

Assuming that there is a normal proportion of granulocytes, the chemotherapy will be allowed to continue if the WBCC is within the range of 3.0 to 4.0 × 10^9^/l. If the WBCC falls within the range of 2.0 to 3.0 × 10^9^/l, chemotherapy will be stopped until the WBCC increases to 3.0 × 10^9^/l, or above. Granulocyte colony-stimulating factor will be used for supportive treatment if WBCC decreases to 2.0 × 10^9^/l or below.

The doses of chemotherapy drugs will be decreased by 25% in the next cycle if IV degree toxicity (including fever-induced neutropenia syndrome) emerges. Chemotherapy will be stopped in advance in cases of disease progression, or unacceptable AEs,,even the patient may not finish the chemotherapy plan.

### Primary and secondary outcome measures

#### Primary

Objective response rate (ORR): total of CR + PR.

Change in regional lymphatic metastasis (including number and size).

Change in TNM staging.

Efficacy will be evaluated every two cycles, with chest CT performed 4 weeks after each evaluation to confirm if the result is CR or PR.

#### Secondary

Progression-free survival.

Objective survival.

Median survival time.

Survival rate at 1 year.

Time to disease progression.

Toxicity.

Follow-up will consist of a telephone survey and a visit to the clinic for re-examination. Chest CT will be performed every 4 months. All patients will be followed up for at least 12 months.

### Reporting of adverse events

Participants will be requested to report any AEs occurring at any point throughout the whole trial, and the doctors will give appropriate advice. All AEs will be recorded in detail by the researchers, using a questionnaire that includes the following.

Description of the AE and associated symptoms.

When did the AE happen?

What was the severity of the AE?

How long did the AE last?

What steps did the doctor or patient take after the AE?

What was the outcome of the AE?

The severity of the AE will be graded using National Cancer Institute Common Toxicity Criteria (NCICTC,3rd edition) for drug-associated AEs. Any AEs not included in the classification will be evaluated using a four-grade scale: I (mild), 2 (moderate), 3 (severe), and 4 (extremely severe, i.e. life-threatening).

Severe AEs will be reported to the local authorities within 24 hours, in accordance with the relevant procedures.

### Sample size

The patient sample size is based on our preliminary study, in which the ORRs of the LP and GP groups were found to be about 65% and 35%, respectively. Using a two-tailed significance level of 5% (*α* = 0.05) and power of 90% (*β* = 0.10), we calculated the sample size according to the following formula:

$$\begin{array}{c} n_{1}=n_{2}\\ \;=\frac{{\left(Z_{\alpha }\sqrt{2\bar{p}\left(1-\bar{p}\right)}+Z_{\beta }\sqrt{p_{t}\left(1-p_{t}\right)+p_{c}\left(1-p_{c}\right)}\right)}^{2}}{{\left(p_{t}-p_{c}\right)}^{2}}, \end{array}$$

where *p*_*t*_ is the objective response rate of the LP group (65%), *p*_*c*_ is the objective response rate of the GP group (35%), and $\bar{p}$ is the average response rate of both groups: (65% + 35%)/2 = 50%. The sample size is then calculated as: *n*_1_ = *n*_2_ ≈ 57.

Allowing a 10% drop-out rate during the study, we have estimated that 63 patients will need to be enrolled in each group, that is, 126 patients in total. Intention-to-treat (ITT) analysis will be applied to minimize bias due to drop-outs.

### Randomization method

Participants who satisfy the inclusion/exclusion criteria will be randomized in a 1:1 ratio using a computer-generated randomization number table at an independent department (Center of Evidence-Based Medicine and Clinical Epidemiology) who will not take part in the following enrollment. To guarantee correct randomization, the patient identification number and treatment allocation will be provided by a central telephone-based randomization system. After the patient's eligibility for enrollment has been assessed, a unique randomization number will be sent to our department through this central randomization system, and the patient will be assigned to the appropriate treatment group, thus each group will be allocated 63 participants. To minimize the possibility of bias in reporting and assessing the endpoints, the trial will use a PROBE (Prospective Randomized Open trial with Blinded Evaluation of outcomes) design.

### Data collection and statistical analysis

Codes will be used to guarantee the privacy of the participants. The CRF will be used to record data for all participants. Basic patient information, results of all examinations, chemotherapy programs used, AEs observed, and follow-up results will be recorded in a timely and truthful manner. These data will be held by both our department and the sponsor, and cannot be modified.

All participants will be enrolled in the final analysis regardless of whether they withdraw at any point. A descriptive statistical analysis will be performed for most of the study variables. The mean, median, standard deviation, maximum, and minimum will be calculated for quantitative variables, and the absolute and relative frequency, ratio, and CI calculated for qualitative and ranked variables.

The statistical analysis will be carried out by a biostatistician at the Center of Evidence-Based Medicine and Clinical Epidemiology, and the data will be analyzed statistically using SPSS software (version 15.0; SPSS Inc., Chicago, IL, USA). The ITT population, which will include all randomized patients, will be used for efficacy analysis. In addition, a per-protocol analysis will be performed on the efficacy endpoint. The ORRs, 1-year survival rate, and toxicity will be compared for the two groups using Fisher’s exact test, and the survival curve will be analyzed using the Kaplan-Meier method and log-rank test. A two-tailed test will be used for all statistics. P < 0.05 will be considered significant, and 95% confidence intervals will be calculated.

## Discussion

Lung cancer is the most common cancer worldwide, with NSCLC accounting for 85% of cases [20]. Over the past 20 years, the 5-year survival rate for lung cancer has remained low (about 15%) [21]. Over half of patients with NSCLC present with regional lymphatic and/or distant metastasis (stage IIIB and IV) at time of diagnosis, highlighting the need for new strategies to improve the prognosis of this disease [7].

Liposomal paclitaxel is a new formulation composed of paclitaxel packaged with liposomes, which has favorable pharmacokinetic properties including significant prolongation of elimination half-life and mean retention time, and an apparently larger volume of distribution *in vivo*. In particular, the dramatically higher drug concentration in the lymph nodes compared with the current paclitaxel formulations indicates that patients with NSCLC with regional lymphatic metastasis may benefit from this new drug. Because doublet chemotherapy composed of cisplatin and gemcitabine has commonly been recommended as first-line therapy for patients with advanced NSCLC [4,22,23], we have designed this open-label RCT to assess whether liposomal paclitaxel plus cisplatin (LP) is superior to gemcitabine plus cisplatin (GP) in efficacy (both short-term and long-term efficacy) and safety (toxicity).

This study will be the first RCT to investigate whether first-line therapy with liposomal paclitaxel can improve the prognosis of patients with NSCLC with regional lymphatic metastasis. The results of this study may provide a new chemotherapy regimen for clinicians and promote new trials to focus on patients with NSCLC with regional lymphatic metastasis.

## Trial status

Currently, 57 patients have been enrolled and randomized for this trial, and recruitment is ongoing.

## Abbreviations

AE: Adverse event; ALT: Alanine aminotransferase; AST: Aspartate aminotransferase; CR: Complete response; CRF: Case report form; CT: Computed tomography; CVD: Cardiovascular disease; ECG: Electrocardiography; GP: Gemcitabine and cisplatin; LP: Liposomal paclitaxel and cisplatin; IV: Intravenous; IM: Intramuscular; ITT: Intention-to-treat; NCICTC: National Cancer Institute Common Toxicity Criteria; NSCLC: Non-small cell lung cancer; ORR: Objective response rate; PR: Partial response; RECIST: Response Evaluation Criteria in Solid Tumor; ULN: Upper limit of normal; WBCC: white blood cell count; WHO: World Health Organization.

## Competing interests

All the authors declare no conflict of interest.

## Authors’ contributions

All authors have made a substantial contribution to this manuscript and study design in regards to conception, design, and drafting. ZXD is the lead investigator, treating physician for the participants, and corresponding author for the manuscript, and has been involved in the trial methodology planning; LH is the local primary investigator involved in the original protocol drafting, data collection, and supervision of the study. GL and WYL are the treating physicians for the participants and are also involved in patient recruitment and treatment; ZBJ is involved in data collection and patient follow-up, and XRF is providing statistical assistance. All authors have read and approved the final manuscript.
